# Prevention of Oxygen Desaturation in a Patient With Previous Experience of Severe Hypoxia in Modified Electroconvulsive Therapy by Transnasal Humidified Rapid-Insufflation Ventilator Exchange: A Case Report

**DOI:** 10.7759/cureus.60564

**Published:** 2024-05-18

**Authors:** Yukari Toyota, Takashi Kondo, Soshi Narasaki, Hirotsugu Miyoshi, Yasuo M Tsutsumi

**Affiliations:** 1 Department of Anesthesiology and Critical Care, Hiroshima University, Hiroshima, JPN

**Keywords:** apneic oxygenation, hypoxia, electroconvulsive therapy, hfnc, thrive

## Abstract

Transnasal humidified rapid-insufflation ventilator exchange (THRIVE) has been reported to have better efficacy during anesthesia induction compared to conventional mask ventilation, including improved oxygenation and prolonged safe apnea time. This study reports on the effectiveness of the THRIVE system during modified electroconvulsive therapy (mECT) for a patient experiencing severe hypoxia. A 78-year-old female patient with bipolar disorder received maintenance mECT every four weeks. She previously experienced a significant hypoxic event, with oxygen saturation (SpO_2_) dropping to 50% following electrical stimulation. In response, we employed the THRIVE system, designed to deliver high-flow, 100% oxygen, thereby extending apnea tolerance. The implementation of THRIVE ensured a stable oxygen supply, maintaining oxygen saturation levels above 95% throughout the mECT procedure. THRIVE is useful for treating hypoxia that occurs due to the unavoidable lack of ventilation during mECT.

## Introduction

The main purposes of anesthesia for modified electroconvulsive therapy (mECT) are to induce unconsciousness that relieves the patient’s anxiety and to prevent physical trauma such as fractures and dislocations associated with seizures by muscle relaxants. Nevertheless, it necessitates respiratory support until the patient regains adequate spontaneous respiration due to the induced apnea. The guideline by the American Psychiatric Association states that anesthesia for ECT should be administered by a specially trained anesthesiologist and that the anesthesiologists have overall responsibility, not only for anesthesia itself but also for cardiopulmonary management and emergency care [1].

Since ECT is a brief procedure lasting only a few minutes, it is performed under general anesthesia with short-acting sedatives and muscle relaxants. The muscle relaxants induce apnea, requiring respiratory support during the procedure. However, during the seizures, manual ventilation is interrupted because the patient cannot be touched. After the end of the seizures, respiratory support is resumed while waiting for the effects of the anesthetic and muscle relaxants to wear off and for spontaneous breathing and consciousness to return.

A standardized approach for airway management in mECT involves manual ventilation through a face mask. However, maintaining sufficient oxygenation may prove challenging due to potential complications, such as issues with mask ventilation or extended periods of apnea during electrical stimulation. Hypoxemia in ECT is often observed and its incidence has been reported to be as high as 29% [2]. It is a complication that should be avoided, not only because it can cause cardiac arrest and hypoxic encephalopathy in severe cases but also because it has been associated with repeated episodes of oxygen deprivation and impaired attention and executive function [3].

Transnasal humidified rapid-insufflation ventilatory exchange (THRIVE) represents a novel technique that delivers warmed, humidified oxygen through a high-flow nasal cannula (HFNC), supplying 100% humidified oxygen at rates up to 70 L/min. This approach significantly extends the duration of safe apnea [4]. Previous reports have demonstrated the effectiveness of THRIVE in preventing desaturation during anesthesia induction and tracheal intubation in patients with difficult airways undergoing general anesthesia. Although recent a few studies [5] have reported the use of THRIVE, its effectiveness in airway management during mECT has not yet been fully established.

In this report, we present a case demonstrating the efficacy of THRIVE in a patient who had encountered severe hypoxia during previous mECT sessions.

## Case presentation

A 78-year-old woman, with a history of bipolar disorder, previously receiving mECT every four weeks for four years at an external hospital, was transferred to our facility for continued mECT treatment. Her pre-treatment evaluation revealed overweight status (height: 156 cm; weight: 71 kg; body mass index (BMI): 29.3 kg/m^2^) and impaired glucose tolerance.

Our hospital adheres to a standardized anesthetic protocol for m-ECT, which involves the following steps: initial preoxygenation for three minutes, followed by rocuronium (4 mg) for precurarization of suxamethonium (40 mg) and thiamylal (100 mg) to induce unconsciousness. Upon verifying loss of consciousness, suxamethonium is administered to facilitate muscle relaxation, concurrently with the initiation of mask ventilation. After confirming that fasciculation in the toes has disappeared, mask ventilation is briefly halted to allow for the electroconvulsive stimulation, administered by a psychiatrist. Ventilation through the mask is resumed immediately after the cessation of seizure activity.

**Figure 1:**
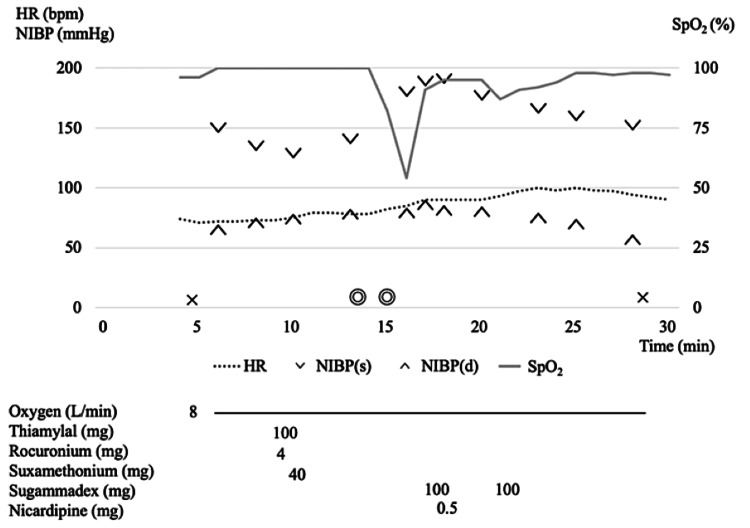
Anesthesia record: without THRIVE SpO_2_ dropped markedly to 50% after electrical stimulation. It improved after mask ventilation was resumed. Considering the possibility that the residual effect of a small dose of rocuronium administered for precuracization may have caused respiratory depression, Sugammadex was administered. HR: heart rate, NIBP(s): systolic blood pressure, NIBP(d): diastolic blood pressure, SpO_2_: percutaneous oxygen saturation, THRIVE: transnasal humidified rapid-insufflation ventilator exchange, ×: the start/end of anesthesia, ◎: the start/end of surgery

The oxygen saturation (SpO_2_) of the patient decreased to approximately 80% following electrical stimulation after each ECT session, beginning with the first. After these episodes, oxygenation consistently improved with the resumption of ventilation. Notably, during the 12th ECT session, despite the administration of the same anesthetic dosage as in previous sessions, the patient experienced a significant drop in SpO_2_ to 50% post-stimulation (Figure 1).

SpO_2_ gradually increased with the application of mask ventilation. Considering the possibility that the residual effects of rocuronium may have caused respiratory depression, we administered sugammadex as a precaution. The patient's postoperative recovery was without complications. In this 12th session, the duration of the seizures was 26 seconds, and the duration of apnea was 120 seconds during this session, which did not differ from the mean seizure duration of 28.4 ± 13.8 seconds and apnea duration of 152 ± 55.4 seconds in the previous sessions.

Given this severe hypoxic incident, it was decided to utilize Optiflow THRIVE (Fisher and Paykel Healthcare Limited, Panmure, Auckland, New Zealand) for the delivery of high-flow 100% oxygen in future sessions to prevent desaturation during electrical stimulation.

**Figure 2:**
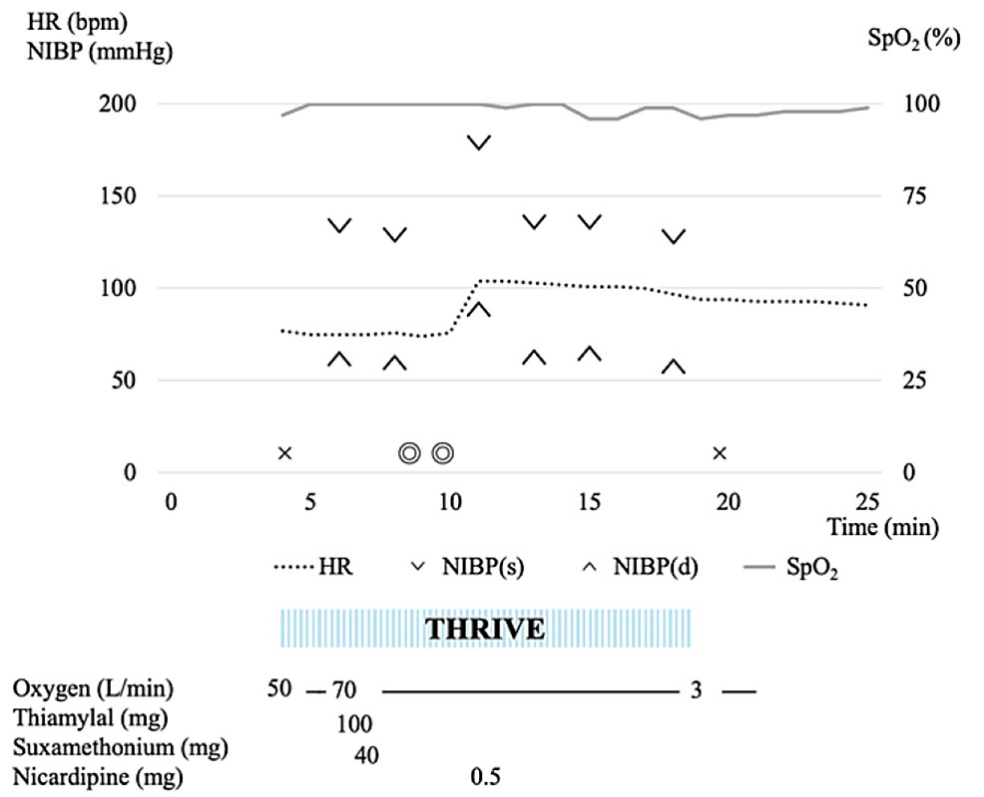
Anesthesia record: with THRIVE SpO_2_ did not decrease after electrical stimulation, and SpO_2_ remained above 95% throughout the entire procedure. HR: heart rate, NIBP(s): systolic blood pressure, NIBP(d): diastolic blood pressure, SpO_2_: percutaneous oxygen saturation, THRIVE: transnasal humidified rapid-insufflation ventilatory exchange, ×: the start/end of anesthesia, ◎: the start/end of surgery

The anesthesia record in mECT using THRIVE is presented in Figure 2.

Upon the patient's entry into the operating room, standard monitoring equipment, including an electrocardiogram, SpO_2_, and non-invasive blood pressure (NIBP), was applied, and baseline vital signs were recorded: HR of 75 bpm, SpO_2_ of 97% (room air), and NIBP of 134/62 mmHg. No specific refusal behavior was observed in this patient when THRIVE was put on. The patient underwent pre-oxygenation with high-flow humidified nasal oxygen at 50 L/min for two minutes using the THRIVE device, after which the flow rate was increased to 70 L/min in preparation for anesthetic administration. Following confirmation of sleep induction with thiamylal (100 mg), suxamethonium (40 mg) was administered, and manual ventilation via a face mask commenced. Precurarization was not performed because the residual effect of rocuronium, even in small doses, could have been one of the causes of the severe hypoxemia. Once fasciculation in the toes disappeared after the administration of suxamethonium, electrical stimulation was carried out by the psychiatrist. Mask ventilation was then not administered until seizures resolved. The electroencephalogram (EEG) of the seizure was recorded for 50 s. Mask ventilation with 100% oxygen was reinstated upon a decline in SpO_2 _post-electrical stimulation, which swiftly returned SpO_2 _to 100%. After waiting for stable spontaneous respiration to resume, the use of THRIVE was terminated by decreasing the oxygen flow while ensuring that there was no decrease in SpO_2_. Thereafter, only face masks were used for oxygen administration. The patient was hemodynamically stable and had a lowest SpO_2_ of 95% during this session.

**Figure 3:**
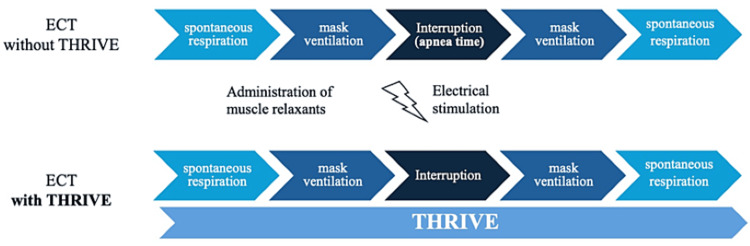
Airway management sequence With THRIVE, apneic oxygenation can be performed during mask ventilation interruption. ECT: electroconvulsive therapy, THRIVE: transnasal humidified rapid-insufflation ventilator exchange

The airway management sequence with and without THRIVE is shown in Figure 3.

## Discussion

Both HFNC therapy and THRIVE provide rapid insufflated heated humidified gases through HFNC, but the common HFNC therapy provides a mixture of air and oxygen at a high flow rate (30-60L/min) to spontaneously breathing patients. The oxygen concentration can be adjusted (FiO_2_ of 0.21-1.0). On the other hand, THRIVE is a method of prolonging safe apnea time and apneic oxygenation in both spontaneously breathing and apneic patients, such as during induction of anesthesia. It can provide 100% oxygen at a flow rate of 70 L/min [6].

THRIVE allowed safe airway management in our patient who experienced hypoxia during each mECT. A previous study identified obesity and seizure duration as independent predictors of hypoxia during recovery from anesthesia during ECT [2]. Our patient demonstrated severe hypoxia, although the seizure duration was similar to that of the previous ECT session, which may be primarily attributed to her obesity. In ECT, a brief but time-consuming period is required not only during electrical stimulation but also during bite block insertion and psychiatrist manipulation of the device, which also prolongs the apneic duration. Although the patient was not difficult to mask ventilate (she was obese), it is possible that inadequate ventilation or lack of mask ventilation for a few seconds could have led to excessive hypoxia. We also considered the possibility that the small dose of rocuronium used for precurarization prolonged respiratory depression. Therefore, precurarization, in which a small dose of a nondepolarizing neuromuscular blocker is used to prevent fasciculation-induced suxamethonium and increased intraabdominal pressure [7], was not performed in the 12th session.

Previous reports have described the efficacy of HFNC therapy in obese patients [8,9]. Wong et al. reported that the use of HFNC in morbidly obese patients during induction of anesthesia resulted in a longer safe apnea time and quicker re-saturation as compared to that observed during conventional facemask oxygenation [8]. HFNC provides a flow rate that exceeds the patient’s peak inspiratory flow rate, resulting in less mixing with ambient air and delivery of a high concentration of oxygen. In addition, flushing deoxygenated air out of the upper airway during expiration ensures delivery of high FiO_2_ levels and further improves ventilatory efficiency by flushing out dead airways [10,11]. HFNC also generates end-expiratory positive airway pressure (PEEP), which may further reduce ventilator load by offsetting automatic PEEP, improving oxygenation, increasing airway patency during expiration, and consequently enabling complete emptying [10]. Notably, Parke et al. reported that, for every 10 L/min increase in flow rate, the mean nasopharyngeal pressure increased by 0.35 cm H_2_O with an open mouth and 0.69 cm H_2_O with a closed mouth [12].

Preoxygenation, followed by apneic oxygenation, is an effective maneuver to prolong the safe apnea time [9]. During regular breathing in adults, approximately 250 mL/min of oxygen flows from the alveoli into the bloodstream, and approximately 250 mL/min of carbon dioxide returns from the bloodstream to the alveoli. However, in apneic patients, approximately 250 mL/min of oxygen flows from the alveoli into the bloodstream, but only 8-20 mL/min of carbon dioxide returns from the bloodstream to the alveoli, with the remainder buffered by the bloodstream and tissues. The difference between the amount of oxygen leaving and the amount of carbon dioxide incoming creates a negative pressure in the alveoli, and if the upper airway is patent, gas will flow in through the pharynx [13]. The administration of supplemental oxygen provides an oxygen-rich mixture to the pharynx, which may in turn improve arterial oxygen saturation. This process is termed apneic oxygenation and permits the maintenance of oxygenation without spontaneous or administered ventilation [13].

The limiting factor for the duration of apneic oxygenation is not oxygenation but rather increased CO_2_ and lower arterial pH [14]. THRIVE differs from conventional apneic oxygen therapy in that it maintains oxygen saturation and reduces carbon dioxide accumulation. Patel demonstrated an average rate of 0.15 kPa/min of carbon dioxide increase with THRIVE [4]. Apneic oxygen therapy with THRIVE is established by a combination of dead space flushing, generation of positive airway pressure, and cardiogenic oscillation while providing high-flow, high-concentration oxygen [15,16]. Hermez et al. reported that high-flow nasal oxygenation produces a flow of gas due to cardiogenic oscillations that transport carbon dioxide from the carina to the pharynx. They further demonstrated that the clearance of carbon dioxide during THRIVE consisted of an interaction between the supraglottic flow vortex produced by high-flow nasal oxygen and the flow oscillations produced by cardiogenic oscillations [15].

Several reports have described the usefulness of THRIVE in ECT [5,17,18]. Jonker et al. performed 20 sessions of ECT with THRIVE on 13 patients and observed no desaturation or patient discomfort during the procedure [17]. Vaithialingam et al. performed a crossover study in which each patient underwent the conventional face mask ventilation and the THRIVE methods in separate ECT sessions [5]. This involved the usage of a cervical collar, which simultaneously provides jaw thrust and cervical spine control in the THRIVE group since the effectiveness of THRIVE depends on the patient having a patent upper airway. The study reported an incidence of 0.5% desaturation with THRIVE, which proves its effectiveness in maintaining oxygenation in patients undergoing ECT while wearing a cervical collar [18]. In these reports, preoxygenation was performed at 30 L/min of oxygen, then increased to 50-70 L/min during induction of anesthesia and maintained throughout the procedure. THRIVE was discontinued when sufficient spontaneous breathing was observed, with oxygen flow reduced by 1-10 L/min after the end of electrical stimulation or resumption of spontaneous respiration. In our case, the patient was obese, which can develop atelectasis, and had experienced severe hypoxia. Thus, preoxygenation was performed at 50 L/min of oxygen and increased to 70 L/min at anesthesia induction and maintained subsequently. After the procedure, we waited for the patient to achieve stable spontaneous breathing then reduced the oxygen flow rate while carefully checking that there was no decrease in SpO_2_, terminated the use of THRIVE, and switched to a face mask. 

In ECT, where apnea during electrical stimulation is unavoidable, it makes sense to use THRIVE, which provides apneic oxygenation. Furthermore, preoxygenation and hyperventilation during ECT are known to improve both safety and seizure quality [19]. Therefore, we postulate that THRIVE can provide more stable airway management and improve ECT quality.

## Conclusions

We used THRIVE for airway management of an obese patient who had repeated hypoxic episodes during ECT. The use of THRIVE successfully completed the entire intraoperative course without hypoxia.

THRIVE allows apneic oxygenation by delivering high-flow nasal oxygen. In ECT, where no ventilation time is inevitable during convulsive stimulation, THRIVE contributed significantly to the maintenance of intraoperative oxygenation not only through preoxygenation but also through apneic oxygenation.

## References

[1] American Psychiatric Association (2001). The Practice of Electroconvulsive Therapy, Second Edition Recommendations for Treatment, Training, and Privileging (A Task Force Report of the American Psychiatric Association). https://www.appi.org/Products/Electroconvulsive-Therapy/Practice-of-Electroconvulsive-Therapy-Second-Editi.

[2] Surve R, Bansal S, Sriganesh K, Subbakrishna DK, Thirthalli J, Umamaheswara Rao GS (2015). Incidence and risk factors for oxygen desaturation during recovery from modified electroconvulsive therapy: a prospective observational study. J Anaesthesiol Clin Pharmacol.

[3] Yamout K, Goldstein FC, Lah JJ, Levey AI, Bliwise DL (2012). Neurocognitive correlates of nocturnal oxygen desaturation in a memory clinic population. J Clin Exp Neuropsychol.

[4] Patel A, Nouraei SA (2015). Transnasal humidified rapid-insufflation ventilatory exchange (THRIVE): a physiological method of increasing apnoea time in patients with difficult airways. Anaesthesia.

[5] Vaithialingam B, Bansal S, Muthuchellappan R, Thirthalli J, Chakrabarti D, Venkatapura RJ (2023). Comparison of hands-free trans-nasal humidified rapid insufflation ventilatory exchange (THRIVE) with conventional facemask ventilation technique for oxygenation in patients undergoing electroconvulsive therapy - a cross over study. Asian J Psychiatr.

[6] Vaithialingam B, Sriganesh K (2023). Trans-nasal humidified rapid insufflation ventilatory exchange (THRIVE) in neuroanesthesia practice: a review. J Anaesthesiol Clin Pharmacol.

[7] Martin R, Carrier J, Pirlet M, Claprood Y, Tétrault JP (1998). Rocuronium is the best non-depolarizing relaxant to prevent succinylcholine fasciculations and myalgia. Can J Anaesth.

[8] Wong DT, Dallaire A, Singh KP (2019). High-flow nasal oxygen improves safe apnea time in morbidly obese patients undergoing general anesthesia: a randomized controlled trial. Anesth Analg.

[9] Baraka AS, Taha SK, Siddik-Sayyid SM (2007). Supplementation of pre-oxygenation in morbidly obese patients using nasopharyngeal oxygen insufflation. Anaesthesia.

[10] Spoletini G, Alotaibi M, Blasi F, Hill NS (2015). Heated humidified high-flow nasal oxygen in adults: mechanisms of action and clinical implications. Chest.

[11] Ritchie JE, Williams AB, Gerard C, Hockey H (2011). Evaluation of a humidified nasal high-flow oxygen system, using oxygraphy, capnography and measurement of upper airway pressures. Anaesth Intensive Care.

[12] Parke RL, Eccleston ML, McGuinness SP (2011). The effects of flow on airway pressure during nasal high-flow oxygen therapy. Respir Care.

[13] Weingart SD, Levitan RM (2012). Preoxygenation and prevention of desaturation during emergency airway management. Ann Emerg Med.

[14] Gustafsson IM, Lodenius Å, Tunelli J, Ullman J, Jonsson Fagerlund M (2017). Apnoeic oxygenation in adults under general anaesthesia using transnasal humidified rapid-insufflation ventilatory exchange (THRIVE) - a physiological study. Br J Anaesth.

[15] Hermez LA, Spence CJ, Payton MJ, Nouraei SA, Patel A, Barnes TH (2019). A physiological study to determine the mechanism of carbon dioxide clearance during apnoea when using transnasal humidified rapid insufflation ventilatory exchange (THRIVE). Anaesthesia.

[16] Lyons C, Callaghan M (2019). Uses and mechanisms of apnoeic oxygenation: a narrative review. Anaesthesia.

[17] Jonker Y, Rutten DJ, van Exel ER, Stek ML, de Bruin PE, Huitink JM (2019). Transnasal humidified rapid-insufflation ventilatory exchange during electroconvulsive therapy: a feasibility study. J ECT.

[18] Vaithialingam B, Bansal S, Ramesh VJ, Muthuchellappan R (2022). Trans-nasal humidified rapid insufflation ventilatory exchange (THRIVE) ventilation during electroconvulsive therapy (ECT) for a pregnant patient - a novel technique. Asian J Psychiatr.

[19] Sawayama E, Takahashi M, Inoue A (2008). Moderate hyperventilation prolongs electroencephalogram seizure duration of the first electroconvulsive therapy. J ECT.

